# Multiple Complications by Hydatid Cyst-induced Budd Chiary Syndrome: A Case Report

**Published:** 2017

**Authors:** Feidoun SABZI, Reza FARAJI

**Affiliations:** Preventive Cardiovascular Research Centre Kermanshah, Kermanshah University of Medical Sciences, Kermanshah, Iran

**Keywords:** Hydatid cyst, Cardiac complication, Budd chiary syndrome

## Abstract

A case of the Budd Chiary Syndrome (BCS) in a 43-yr-old man with hydatid cyst (HC) in the liver is reported from Imam Ali Hospital, Kermanshah, western Iran in 2016. This case was complicated by inferior vena cava (IVC) thrombosis, right atrial clot and pulmonary emboli. Compression of IVC was the likely mechanism. Abdominal ultrasonography revealed a huge HC located in segments near IVC and caused compression of IVC. Transthoracic echocardiography (TTE) revealed IVC and right atrium thrombosis, however pulmonary emboli was not documented in TTE but intra operative exploration showed multiple clot in main and left pulmonary artery branch. The patient recovered after open-heart surgery with removal of right atrial, IVC and pulmonary artery emboli. BCS should be looked for routinely in patients with HC of the liver.

## Introduction

Hydatid cyst (HC) is a larval tape-worm infestation that occurs in humans after ingestion of foods contaminated with the larval form of *Echinococcus.* It is one of the most dangerous zoonotic diseases in the world. HC most commonly invades the liver, and may lead to invasive and destructive changes in peri cyst organs (1).

HC rarely causes budd chiary syndrome (BCS) because of occlusion of the hepatic veins and IVC (1), involvement of the great vessels by HC of the liver and because lung is not common and usually involves the thoracic aorta by invasion of cyst from neighboring organs. Another mechanism is infestation of the great vessel wall directly by blood circulation. This mechanism of larva nesting theoretically is reported in the aortic wall with its rich vaso vasurum. In some case larva penetrates directly from circulation to endothelium and media of vessel. In opposing to the aorta, great veins wall have not vaso-vasurum and inferior vena cava (IVC) were not directly affected by blood borne hydatid larva but compressed by HC. Great vein complications are rare, generally involving superior vena cava syndrome (SVC) or BCS (2).

We report a rare case of BCS complicated by IVC, RA and PA thrombosis.

## Case report

A case of the budd chiary syndrome (BCS) in a 43-yr-old man with hydatid cyst (HC) in the liver is reported from Imam Ali Hospital, Kermanshah, western Iran in 2016. He was presented with abdominal distention, lower extremity edema and severe dyspnea. Informed consent was taken from the patient.

**Fig. 1:**
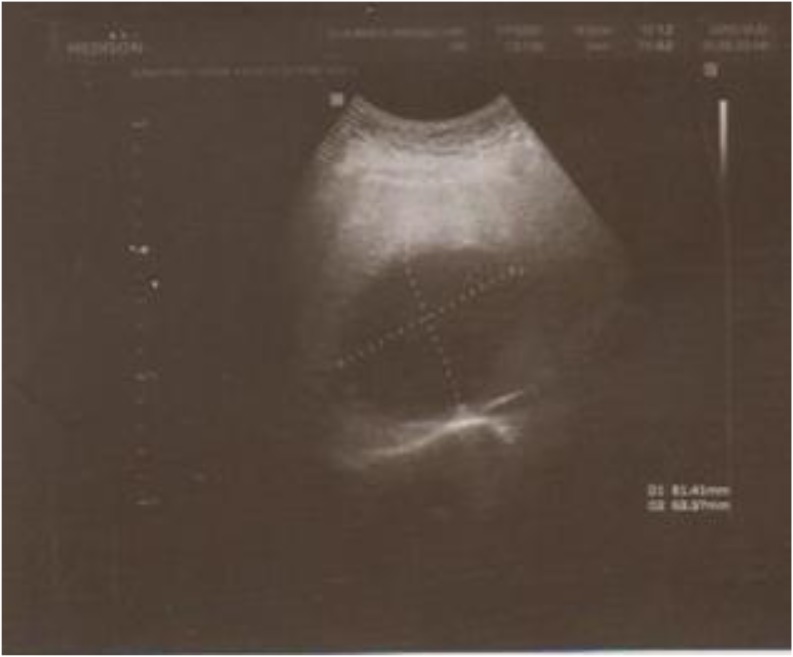
Abdominal ultrasound shows large hydatid cyst compressing inferior vena cava

On physical examination, tension ascites but no any collateral veins were noted on the abdomen. Respiratory sounds were normal at the bases of both lungs. The abdominal ultra-sound revealed venous outflow obstruction caused by HC occupying supra hepatic veins of an IVC segment of the hepatic dome and measuring 8 cm on average (Fig. 1). There was no flow viewed in the left hepatic vein and IVC on abdominal portal Doppler ultrasound. The portal vein was 20 mm, and the splenic vein was 18 mm in diameter and hepatofugal flow was noted.

**Fig. 2:**
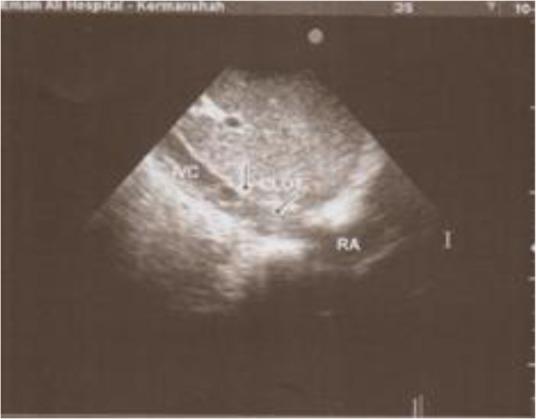
Echocardiography shows thrombosis in inferior vena cava extended to right atrium

It was occluding the IVC and left hepatic vein at the level where the hepatic veins poured into the IVC. TTE also revealed a large thrombosis in the right atrium that came out from IVC orifice. The thrombosis was mobile however, no any clot was found in the main pulmonary artery or its left and right major branches (Fig. 2). With para centhesis, the intra peritoneal fluid was not clear and total leukocyte count was, 90% of lympho-monocyts. The intra peritoneal fluid was exudates with high albumin (0.6 g/dl) and protein 5.6 g/dl. There was no other pathology found in the Gram staining and culture of the intra peritoneal fluid. According to these findings, the patient was diagnosed with secondary (BCS) developmental due to HC.

**Fig. 3:**
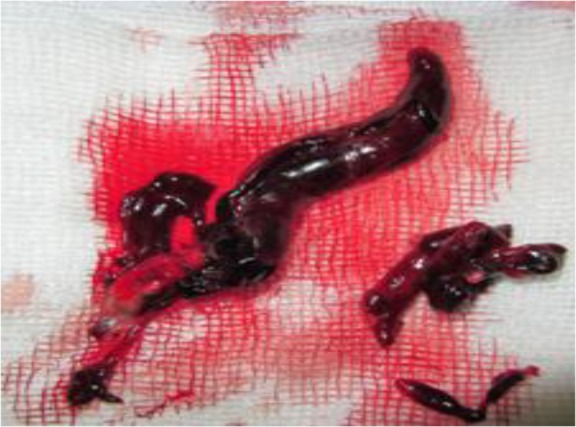
Shows long segment thrombosis extracted from inferior vena cava

The patient scheduled for emergency removal of right atrial clot and possible pulmonary embolectomy. The standard median sternotomy was carried out and CPB was constituted by the aorta, SVC and femoral vein cannulation. Following cardiac arrest by cardioplegin, right atrium and main pulmonary arteries were opened. A long segment of the clot was removed from IVC as a small piece of clot suctioned from the left pulmonary artery (Fig. 3). The IVC was repeatedly suctioned to sure that no thrombosis was remained in its wall and its accessory branches. Surgical treatment successfully restored venous out-flow in our patient. The operation had an uneventful postoperative course and the patient discharged on the 14^th^ day of hospitalization and referred to a general surgery center for the management of his liver HC.

## Discussion

Intermittent or temporary obstruction IVC outflow tract in supra hepatic veins is reported in very rare case of BCS. Unknown osmotic change of intra hydatid fluid or peri cystic edema or leaking of cyst fluid to neighboring organs and abscess formation may make larger the size of the cyst and ended to obstruction of IVC. The obstruction by voluminous cyst also may involve both hepatic vein outflow and main IVC trunk in supra hepatic vein location. The most common cause of BCS is lung carcinoma. Many others causes have been described. The second predominant cause is thrombophilias (3).

The prevalence of underlying thrombophilias including hereditary and acquired hyper-coagulable states are markedly increased in patients with BCS. Third common factor is myeloproliferative syndromes, myeloproliferative disorders are found to be the leading cause of BCS in western countries, with a range between 20%–53%. Congenital webs are common and the most easily treated lesions in some studies (16%). The other less common cause is a coagulation factor deficiencies, and antiphospholipid syndrome. Hydatid cysts are the exceedingly rare cause of BCS (4).

In East Asia, staphylococcus infection of IVC endothelium, amebic abscess are common etiologies of BCS. Another frequently observed etiology has been Behcet’s disease with a ratio of 9% (4). The etiology of BCS is the most important factor for the prognosis. In some cases, invasion of cyst to neighboring area causes severe inflammation, infection and fistula formation. In addition to local invasive effect of cyst, hepatic dysfunction is another’s important prognostic risk factor for surgical resection outcome.

Mortality and morbidity of surgical treatment for hydatid cysts of the liver in patients with BCS is 8.3% and 66% subsequently (5). The thick inflammatory process around of HC not only complicated safe resection of cyst or associated liver segment but abscess and inflammation of tissue prohibits easily suture of injured biliary tract during operation and increased postoperative biliary fistula.

Karadas et al. reported a low-gradient ascites in connection with the syndrome that rarely occurs in case of HC of the liver (6). Hydatid disease should be considered in the differential diagnosis of Budd-Chiari Syndrome in areas such as Turkey, where hydatid disease is endemic (7). HC of the left lobe of the liver has potential to extend to the posterior mediastinum and invade the diaphragm, esophagus, and pericardium (8). The cystic lesion was seen to be occluding the inferior vena cava and left hepatic vein at the level where the hepatic veins poured into the inferior vena cava.

We discussed this secondary BCS case, resulting from the AE occlusion of inferior vena cava. BCS and portal hypertension is an uncommon complication of hydatid cyst of the liver. Çakmak et al. described the treatment of BCS with resection of the cyst or porto-systemic shunt surgery for such patients (8).

Here we reported a case of hydatid cyst of the liver with temporary BCS that was treated successfully with surgical removal of IVC clot and anticoagulant therapy.

Amoebic liver abscess is an endemic in developing countries, but few cases of associated vascular complications have been reported (9). Agarwal and Kumar reported a 13-yr-old girl presented with two large unilocular liver hydatids complicated by chronic BCS (10). Rossi et al., presented a case of recurrent inferior vena cava (IVC) syndrome due to alveolar echinococcosis and strongly suspected on transthoracic echocardiographic examination (11). Emre et al., reported two cases of portal hypertension due to hydatid cysts of the liver (12). In one of the patients, symptoms were secondary to obstruction of inferior vena cava and hepatic outflow tract.

## Conclusion

Presence of clot in right atrium may be a rare presentation of BCS.
